# Bacterial Communities Associated With Acute Oak Decline of Sessile Oak ( *Quercus petraea* ) in Southern Sweden

**DOI:** 10.1111/1758-2229.70244

**Published:** 2025-11-21

**Authors:** Dániel G. Knapp, Johanna Sunde, Meysam BakhshiGanje, Johanna Witzell

**Affiliations:** ^1^ Department of Forestry and Wood Technology Linnaeus University Växjö Sweden; ^2^ Centre for Ecology and Evolution in Microbial Model Systems, EEMiS, Department of Biology and Environmental Science Linnaeus University Kalmar Sweden; ^3^ Kohgiluyeh va Boyer‐Ahmad Agricultural and Natural Resources Research and Education Center Yasouj Iran

**Keywords:** *Enterobacteriaceae*, forest pathology, *Gibbsiella*, *Lonsdalea*, *Pectobacteriaceae*, third generation sequencing, tree microbiome

## Abstract

Acute oak decline (AOD) is a rapidly progressing disease affecting various oak species (*Quercus* spp.). Recent studies have shown that AOD is associated with a consortium of Gram‐negative, facultatively anaerobic bacteria (e.g., in Enterobacterales) in the United Kingdom and continental Europe. However, there is limited information on the bacterial contributions and key genera associated with oak diseases and broadleaf forest ecosystems in Nordic countries. The primary objective of this brief study was to collect the first data on the bark microbiomes of symptomatic, declining sessile oaks ( *Q. petraea* ) in Sweden. Pairs of healthy and diseased bark samples were collected from symptomatic trees near Ankarsrum (Kalmar County), Sweden. After total DNA extraction, the bacterial 16S rDNA region was amplified, and Oxford Nanopore Technologies was used for long‐read high‐throughput DNA metabarcoding of the bacterial microbiome. We found a dominance of enterobacterial phytopathogens, including two of the typical genera associated with AOD, *Brenneria* and *Rahnella*, exclusively in the diseased bark samples. Our findings extend the known distribution of AOD‐associated bacteria to Sweden and Scandinavia and show that diseased oaks in this region host a microbiome similar to those found in other parts of Europe.

## Graphical abstract

Our findings, derived from long‐read high‐throughput DNA metabarcoding of the bacterial microbiome, extend the known distribution of acute oak decline (AOD)‐associated bacteria to Sweden and Scandinavia and show that diseased sessile oaks in this region host a microbiome similar to those found in other parts of Europe.

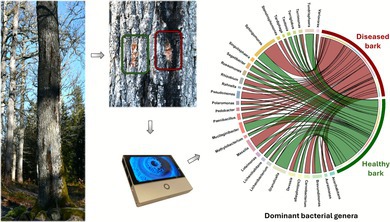

## 1 Introduction

Acute oak decline (AOD) is a rapidly developing disease affecting various oak species (*Quercus* spp.) in Europe (Gosling et al. 2024). It is characterised by deep necrotic lesions in the inner bark and the exudation of copious dark fluid (stem bleeding) from vertical bark fissures (Bene et al. 2025; Gosling et al. 2024). AOD is known as a multifactorial disease driven by a complex interplay of various stress factors, such as prolonged drought, heat stress, and other abiotic conditions, weakening host trees, whilst xylophagous insects and microbial pathogens contribute to the onset and progression of the disease (Gosling et al. 2024). Recent studies showed that AOD is associated with a consortium of gram‐negative, facultatively anaerobic bacteria (mainly in the Enterobacterales) that produce various plant cell wall‐degrading enzymes (Brady et al. 2017; Denman et al. 2018). The key taxa generally identified in symptomatic oak bark are *Brenneria goodwinii*, *Gibbsiella quercinecans* , and *Rahnella victoriana*, with *Lonsdalea britannica* occasionally occurring in some outbreaks (Bene et al. 2025; Maddock et al. 2023). *Brenneria* and *Gibbsiella* are consistently the most abundant AOD‐associated bacteria in Europe, whilst *Rahnella* can also be common in some parts of Europe (Bene et al. 2025).

AOD was first documented in England in the mid‐2000s and has since been reported in various European countries. Recent studies have detected AOD bacterial complexes on *Q. robur* and *Q. petraea* in several European countries including Switzerland, Spain, Portugal, Latvia, and Poland. For instance, *B. goodwinii*, *G. quercinecans* , and *R. victoriana* were first found in 2017 on oaks in Switzerland and Spain, and similar infections have been recorded in central and eastern Europe by 2025 (Bene et al. 2025). The healthy oak‐associated bacteriome generally lacks typical bacterial indicators. Aside from the absence of taxa in the family Enterobacterales, such as *Brenneria* from the tree trunk (Meaden et al. 2016), the dominance of certain taxonomic groups, such as *Pseudomonadaceae* or Pseudomonadota, can be observed in some cases (Brady et al. 2017; Downie et al. 2025). Recent evidence suggests that members of the AOD pathobiome are widespread in oak systems and are likely opportunistic, infecting stressed hosts rather than acting as rare, obligate pathogens (Brady et al. 2017; Denman et al. 2018). Globally, the rapid emergence of slowly proceeding oak declines has been associated with both abiotic stress (Marques et al. 2025; Novick et al. 2022) and pathogens such as *Phytophthora* (Balci and Halmschlager 2003; Jung et al. 2000, 2002). *Phytophthora* infections have been found in declining oak stands in Sweden as well, where *Q. robur* is the most common oak species (Sonesson and Drobyshev 2010).

A recent study (López‐García et al. 2025) revealed significant differences in bacterial alpha diversity between the rhizospheres of healthy and declining *Q. robur* trees in Southern Sweden. However, there is generally limited information on bacterial contributions and key genera associated with oak diseases and broadleaf forest ecosystems in Nordic countries, where forest health is increasingly threatened by the speed and magnitude of climate change (Gauthier et al. 2015). The potential for future northward range expansion of broadleaved forest species such as oaks (Franzén et al. 2024) may be jeopardised by the warming climate and altered precipitation patterns which are creating favourable conditions for bacterial diseases (Kumar and Mukhopadhyay 2024). This highlights the urgent need to better understand and monitor bacterial communities in declining trees.

In this pilot study, we provide the first data on bark microbiomes of declining, AOD‐symptomatic oaks ( *Q. petraea* ) in southern Sweden. We were particularly interested in screening for the presence of AOD bacteria. To our knowledge, this is the first study to document the oak‐bark‐associated bacterial consortium in this region.

## 2 Experimental Procedures

### 2.1 Field Site and Bark Sampling

**FIGURE 1:**
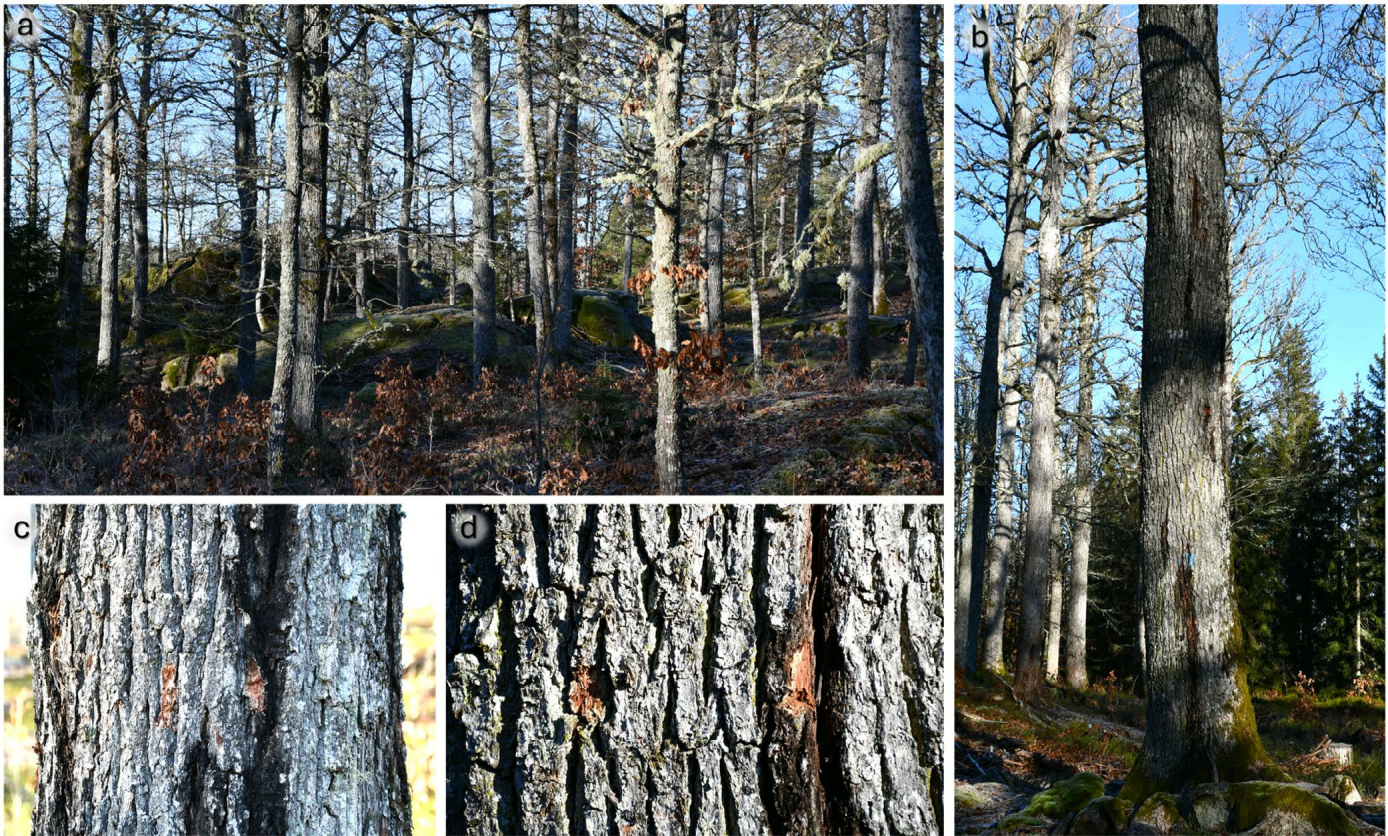
The sampling site and the bark samples. (a) The sessile oak ( *Q. petraea* ) forest near Ankarsrum (Sweden). (b) A tree (Tree_2) showing AOD symptoms and stem bleeding parts, (c) A pair of samples (Pair01) from healthy (on the left) and diseased (on the right) parts of a sampled tree (Tree_1), (d) Another pair of samples (Pair07) from healthy (on the left) and diseased (on the right) parts of a sampled tree (Tree_2).

Samples were collected in a sessile oak ( *Q. petraea* ) forest (57°40′27.0″ N 16°20′59.0″ E) near Ankarsrum (Västervik Municipality, Kalmar County, Sweden) in February 2025. For this, three mature tree individuals that exhibited multiple bleeding parts on the bark were chosen and sampled for four diseased and four healthy bark areas each (Figure 1). Bark samples were collected (using sterilised scissors) from the bleeding zones and from the adjacent healthy‐looking bark; therefore each “diseased” sample was paired with a “healthy” one (Figure 1). For each sample, the outer bark was removed to approximately 1 cm depth at lesion margins, including the region where AOD‐associated bacteria are primarily localised. Bark samples were collected at heights of approximately 150 cm for Tree_1, 150–200 cm for Tree_2, and 50–100 cm for Tree_3, depending on lesion position. No active bleeding or exudate was present; only black discolorations were observed. Each bark sample was placed in a separate plastic bag for transportation to the laboratory. Two of the trees exhibited similar diseased parts and phenotypical features with dark bleeding patches and symptomless parts close to these. The third tree (Tree_3) appeared to be significantly more damaged, with only minor differences between diseased and healthy parts, and with sections of bark slightly detached from the woody tissues near the base of the trunk, suggesting that some lesions may overlap with other superficially similar types of damage potentially caused by multiple factors. We did not observe any evidence of *Agrilus biguttatus* attack, although this insect may play a major role in AOD (see Gosling et al. 2024) and has been documented in southern Sweden, including areas around the sampling site (GBIF occurrence data, accessed 2025‐10‐29). Altogether, we collected four pairs of healthy and diseased samples from each of the three trees, resulting in a total of 24 samples (4 × 2 × 3). Lichens (if present) and other non‐bark particles and tissues were eliminated carefully from the bark samples, which were subsequently washed with 70% ethanol for 30 s, rinsed with sterile distilled water 3 times, and frozen at −20°C. Frozen samples were lyophilized using a freeze dryer (ScanVac CoolSafe, Labogene, Denmark) after 2 days in a freezer. The lyophilized samples were first roughly ground with an electric grinder and further homogenised using a Retsch Mixer Mill MM 400 (Retsch GmbH, Germany).

### 2.2 DNA Extraction and PCR Amplification

For DNA metabarcoding of the bacterial microbiome, total DNA was extracted from approximately 0.2 g of lyophilized and homogenised bark tissue from each of the 24 samples using the DNeasy PowerLyzer PowerSoil DNA isolation kit (Qiagen, Germany) according to the manufacturer's guidelines. The concentration of DNA extracts was measured by fluorometric quantification with the Qubit 4 Fluorometer (Invitrogen, USA) using the Qubit dsDNA high sensitivity assay kit (Invitrogen, USA) following the manufacturer's protocol. The extracted DNA was then diluted to obtain equimolar concentrations, and 10 ng of total DNA from each sample was used for amplification. The bacterial 16S rDNA region was amplified using the tagged universal primers 27F and 1492R included in the 16S Rapid Barcoding Kit 24 V14 (SQK‐16S114.24, Oxford Nanopore Technologies, UK), which was designed for efficient multiplexed full‐length 16S rRNA amplicon sequencing using a protocol that combines the amplification and barcoding of the full‐length 16S rRNA gene in a single PCR step. The 16S rDNA amplification was carried out according to the amplicon sequencing protocol using LongAmp Hot Start Taq DNA Polymerase (Oxford Nanopore Technologies, UK). The purified amplicons were confirmed by visualisation on a 1.5% agarose gel stained with SYBR Green (Thermo Fisher Scientific, Lithuania) and amplicon concentrations were measured by fluorometric quantification with the Qubit 4 Fluorometer similarly to the DNA extracts. The 24 amplicons were diluted and set to have the same amount of DNA in all 24 samples required for the subsequent library preparation.

### 2.3 Library Preparation, High‐Throughput Sequencing, Data Processing and Analyses

Amplicon library preparation for the 24 samples was carried out with the SQK‐16S114.24 16S Barcoding Kit using 10 ng amplicon DNA per sample and according to the V14—amplicon sequencing protocol (Oxford Nanopore Technologies, UK). The amplicon sequencing was performed on a FLO‐MIN114 flow cell on a MinION MK1C device using MinKNOW software version 24.02.16. The minimum and maximum read lengths were set to 500 and 2500 bp, respectively, with a minimum Q score of 15. Sequencing was then run for 72 h. Fast5 files were base‐called with the high‐accuracy model, demultiplexed, and converted to FASTQ format using Guppy v6.1.5. The passed reads were processed using the EPI2ME 16S workflow (Oxford Nanopore Technologies, UK). Taxonomic classification was performed using Kraken2 (v2.1.2) (Wood et al. 2019; Lu et al. 2022) with the NCBI 16S/18S microbial database using default k‐mer settings and using read length 1300–1600 bp, and a minimum Q score of 15. For re‐estimation of abundances Bracken v2.6.2 (Lu et al. 2017) was used with a read length threshold of 1000 bp and a minimum of 10 reads per taxon. The subsequent statistical works and analyses were performed based on the rarefied genus count table obtained from the Bracken analysis. This table comprised the set of 34,695 bacterial 16S sequences per sample based on the one with the fewest reads. One sample out of the 24 (from a diseased part of the third tree) was excluded from further analyses due to its low read number (1146). To make initial functional predictions about prokaryotic communities based on the 16S rRNA amplicon data, we used Tax4Fun2 (Wemheuer et al. 2020), which relies on previously annotated bacterial genomes and the KEGG database for functional annotation, providing outputs in the form of KEGG orthologs (KOs). Raw sequence reads are available in the NCBI Sequence Read Archive under BioProject ID PRJNA1299142. Artificial intelligence assistance (OpenAI) was employed for language editing of the manuscript.

### 2.4 Statistical Analysis

The statistical analysis mainly focused on comparing the data from the diseased and healthy sample pairs (Tree_1 and Tree_2). For documentation, we also reported and analysed the data from the third tree, but these data were treated as two additional distinct sample groups. All statistical analyses were conducted in the R environment for statistical computing (R Core Team 2020). Analysis of variance (ANOVA), non‐metric multidimensional scaling (NMDS), and post hoc tests were performed using R packages vegan (Oksanen et al. 2013), ggplot2 (Wickham 2016), tidyr and dplyr (Wickham et al. 2019). For variance analysis of multiple samples, one‐way ANOVA with Tukey's HSD tests was applied for illustrative purposes, acknowledging that in the case of relative abundance data, the community is compositional and may violate certain assumptions. The results were visualised as boxplots using the ggplot2 package in R (Wickham 2016). Differences in bacterial community composition amongst samples were visualised using non‐metric multidimensional scaling (NMDS) based on Bray–Curtis dissimilarity, applied to a Hellinger‐transformed genus abundance table. Chord diagrams were generated using the circlize package (Gu et al. 2014).

## 3 Results

In this study, long‐read 16S rRNA sequencing, performed on a MinION Mk1c platform (Oxford Nanopore Technologies), yielded a total of 2,458,113 bacterial 16S rRNA reads. These were randomly subsampled for downstream statistical analyses, with 34,695 reads per sample resulting in 797,985 total reads across all samples representing 801 bacterial genera (Table S1).

**FIGURE 2:**
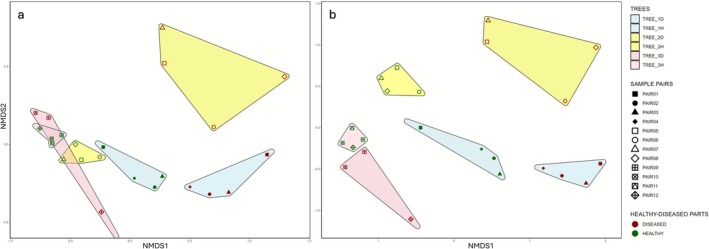
Non‐metric multidimensional scaling (NMDS) plots showing differences in bacterial communities amongst bark samples from healthy (green) and diseased (red) oak trees ( *Quercus petraea* ). (a) NMDS based on genus‐level read counts (Stress = 0.046; PERMANOVA: *R* ^2^ = 0.129, *F* = 3.11, *p* = 0.005). (b) NMDS based on predicted functional profiles (KEGG Orthology abundances; Stress = 0.085; PERMANOVA: *R* ^2^ = 0.114, *F* = 2.71, *p* = 0.005). The analyses were based on Bray–Curtis dissimilarity applied to a Hellinger‐transformed abundance matrices. Each point represents a sample, with symbol fill colours denoting tree identity (Tree 1–3) and point shapes indicating sample pairs. Convex hulls outline groups according to tree identity.

Non‐metric multidimensional scaling (NMDS) was performed on both bacterial genus‐level read counts and predicted functional profiles (KEGG Orthology abundances). In both ordinations, samples clustered according to bark health status (healthy vs. diseased) and tree identity (Trees_1–3), with the exception of the diseased samples from Tree_3, which showed inconsistent grouping (Figure 2). Stress values indicated a reliable ordination of the underlying distance matrices (genus‐based: stress = 0.046; KO‐based: stress = 0.085). PERMANOVA (Bray–Curtis dissimilarities) confirmed significant differences between healthy and diseased bark for both datasets (genus‐based: *R* ^2^ = 0.129, *F* = 3.11, *p* = 0.005; KO‐based: *R* ^2^ = 0.114, *F* = 2.71, *p* = 0.005), whilst tree identity explained an even larger proportion of the variation (genus‐based: *R* ^2^ = 0.424, *F* = 4.03, *p* = 0.001; KO‐based: *R* ^2^ = 0.400, *F* = 3.51, *p* = 0.001). Paired healthy–diseased samples did not show strong clustering. Beta‐dispersion analysis revealed some heterogeneity in group variances (genus‐based: *F* = 7.59, *p* = 0.012; KO‐based: *F* = 7.15, *p* = 0.014), suggesting that both health status and tree‐specific effects contributed to the observed community patterns.

**FIGURE 3:**
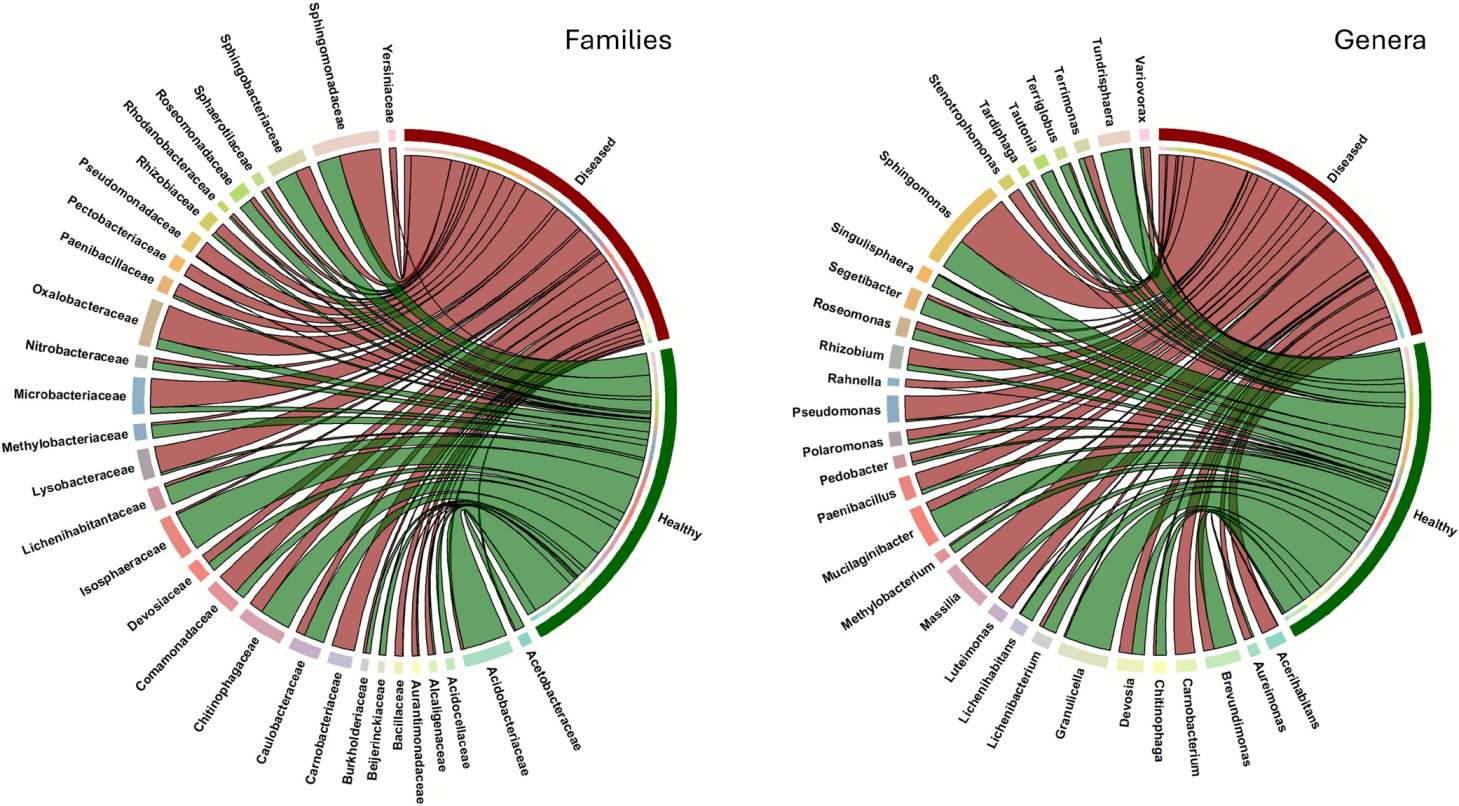
Chord diagrams illustrate the distribution of the top 20 most abundant bacterial families and genera between the eight healthy and eight diseased oak bark samples. The taxa were selected based on their combined relative abundance across both groups. The width of each chord represents the proportion of each genus in the respective group, calculated as percentage of total abundance. Segments in dark green and dark red represent healthy and diseased bark samples, respectively, whilst individual families (on the left) and genera (on the right) are shown with unique colours.

Healthy bark samples exhibited a relatively balanced community structure, with several taxa potentially significantly more abundant within these samples (Figure S1; Table S2). Even at the phylum level, more taxa (Bacteroidota, Acidobacteriota, Planctomycetota, Cyanobacteriota, Armatimonadota, Abditibacteriota, and Gemmatimonadota) were characteristic of healthy tissues, whereas diseased samples were dominated mainly by phylum Bacillota (kingdom Bacillati). In healthy bark tissues, the families *Acidobacteriaceae* and *Isosphaeraceae* and their respective genera *Granulicella* (*Acidobacteriaceae*) and *Tundrisphaera* (*Isosphaeraceae*) were the most common and dominant taxa (Figure S2). Diseased bark showed a clear dominance of several taxa with potentially higher relative abundance compared to healthy bark (Table S2). The genera *Brenneria* and *Rahnella* were consistently present in diseased bark (Figures 3 and S2). In contrast, *Gibbsiella* was detected only at low abundance in a single diseased sample, and *Lonsdalea* was not detected in any sample (Table S1). Notably, members of the Enterobacterales (including *Brenneria*, *Rahnella*, *Erwinia*, *Yersinia*, *Serratia*), the phylum Bacillota (classes Bacilli and Gammaproteobacteria), the orders Burkholderiales, and Micrococcales, the families *Erwiniaceae*, *Enterobacteriaceae*, *Microbacteriaceae*, and *Enterococcaceae*, were more abundant in diseased tissue (Figure 3; Table S2; Figure S2). However, these compositional shifts did not affect standard alpha diversity metrics (Shannon diversity, Fisher's alpha, observed total richness, and Pielou's evenness). No significant differences were detected between healthy and diseased bark samples (ANOVA, Tukey's tests, *p* > 0.05; Table S2), indicating that despite the compositional changes, diversity levels remained similar.

## 4 Discussion

Our study is a pilot investigation and, although it provides important baseline insights into AOD‐associated bacterial communities, it is based on a limited number of trees and samples. Therefore, it has statistical limitations, and the conclusions should be interpreted on an appropriate scale. However, our results clearly indicate that both the taxonomic composition and the predicted functional profiles of bacterial communities are significantly shaped by tree identity and bark health status. The occurrence of *Brenneria* and *Rahnella* only in the diseased oak bark from Sweden is consistent with findings from acute oak decline in the United Kingdom and continental Europe (e.g., Bene et al. 2025; Brady et al. 2017; Denman et al. 2018; Gathercole et al. 2021; Maddock et al. 2023; Sapp et al. 2016; Tkaczyk and Sikora 2024). These two genera, together with *Gibbsiella*, generally dominate lesion margins of diseased oak trees (Denman et al. 2018). Numerous studies have shown that *Brenneria* and *Rahnella*, primarily *B. goodwinii* and *R. victoriana*, are consistently associated with AOD lesions, with *B. goodwinii* usually being the most abundant (Brady et al. 2022). Interestingly, *Gibbsiella* was rare in our samples, since this genus and *G. quercinecans* are also typically common in outbreaks in the United Kingdom (Brady et al. 2017; Maddock et al. 2023). The sporadic or absent detection of the genus *Lonsdalea* and *L. britannica* here corresponds with other studies reporting that *Lonsdalea* is only occasionally isolated in AOD cases (see Bene et al. 2025).

The bacterial taxa enriched in diseased oak bark mainly belong to groups with plant‐pathogenic or saprotrophic lifestyles. Members of Enterobacterales, which include genera such as *Brenneria*, *Erwinia*, *Yersinia*, and *Serratia*, are known plant‐associated bacteria (Kvitko et al. 2025). Their enrichment, along with overall increases in the abundance of Gammaproteobacteria, and the presence of *B. goodwinii* and *G. quercinecans* —species that secrete cell wall‐degrading enzymes that kill host tissue (Doonan et al. 2019)—suggests a shift towards a necrotrophic community in the lesions. The healthy bark harboured a more balanced bacterial community, composed mainly of taxa generally present in environmental samples of forests and dead plant tissues. This suggests that the disease‐associated taxa outcompete the natural microbiota without reducing total bacterial richness.

The fact that we did not observe significant differences in alpha diversity supports the view that AOD causes a shift in community composition rather than a loss of diversity. Sapp et al. (2016) also reported no significant difference in the overall bacterial communities between healthy and diseased trees, and although Brady et al. (2017) found differently diverse bark inhabiting bacterial communities at each investigated site, the diversity of bacteria from the AOD‐associated and healthy tissues was not consistent. They found three genera, *Pseudomonas*, *Halomonas* and *Shewanella* in 80% of healthy bark samples. In these studies from the UK, Gram‐positive families and *Pseudomonas* species were identified as important members of the healthy oak microbiome based on both sequencing and culturing approaches (Brady et al. 2017). In our study, *Halomonas* and *Shewanella* had low abundance and were present only in two diseased samples and in one healthy sample, respectively. Interestingly, *Pseudomonadaceae*, which are generally associated with healthy bark (Brady et al. 2017; Sapp et al. 2016), were more dominant and significantly more abundant in diseased tissues of *Q. petraea* in Sweden. However, Denman et al. (2018) also found that *Pseudomonas* species occurred in both healthy and diseased oak trees. The high abundance of *Pseudomonadaceae* in diseased bark may reflect both opportunistic colonisation and potential antagonistic interactions, as some *Pseudomonas* spp. are known to produce antimicrobial compounds that may influence microbial community dynamics (Haas and Défago 2005), suggesting that functional shifts in the microbiome could modulate disease progression. Our results support the view that these AOD‐associated bacteria are opportunistic and can colonise stressed oaks across different geographic regions, rather than being strictly host‐specific pathogens.

In conclusion, this pilot study represents the first record of AOD‐associated bacterial communities in Sweden. The oak trees sampled here are located around the northern edge of the known range of AOD. Hence, the presence of *Brenneria* and *Rahnella* and other known taxa connected to AOD indicates that these pathogens are already present in southern Sweden and can also be detected in the dormant season. However, it should be noted that our samples were collected only in winter, and microbiome composition and activity may differ during the growing season, when higher temperatures and humidity can enhance microbial colonisation and dynamics. Our findings extend the known distribution of AOD‐associated bacteria into Sweden and Scandinavia and show that diseased oaks in this region may host microbiomes similar to those found in the UK and continental Europe. A more detailed understanding of the bacterial taxa will require additional samples and complementary approaches, including culture‐based isolations and whole‐genome sequencing, and future work should also integrate analyses of fungal communities and *Phytophthora* species alongside bacteria to provide a more comprehensive and realistic picture of oak decline. This knowledge may be important for improving our understanding of forest health in northern Europe under changing climate conditions where oak species may have a more important and increased role in the forthcoming decades.

## Author Contributions

Conceptualization: D.G.K., J.S., M.B., J.W. Methodology: D.G.K., J.S. Formal analysis: D.G.K. Visualization: D.G.K. Data curation: D.G.K. Resources: J.W. Funding acquisition: J.W. Writing – original draft: D.G.K. Writing – review and editing: D.G.K., J.S., M.B., J.W.

## Conflicts of Interest

The authors declare no conflicts of interest.

## Supporting information


**Figure S1:** Column plots show relative abundance of healthy and damaged samples per oak tree individual at different taxonomic levels. The plots show the 30 most abundant taxa and all the others referenced as the group “Other”.

**Figure S2:** Boxplots showing the relative abundances of selected bacterial taxa across the four oak bark sample groups: healthy bark samples (light green), diseased bark samples (pale red), healthy bark tissues of Tree_3 (dark yellow), diseased bark tissues of Tree_3 (dark orange). Each plot represents one bacterial taxon, with individual data points overlaid on boxplots. Statistical differences amongst groups were assessed using one‐way ANOVA followed by Tukey's honest significant difference (HSD) post hoc test. Different letters above the boxes indicate statistically significant differences between groups (*p* < 0.05).

**Table S1:** Genus abundance table comprising rarefied read numbers and taxonomic assignments.

**Table S2:** Taxa associated with healthy and damaged bark tissues, and the related statistics on diversity and differences amongst sample groups at different taxonomic levels.

## Data Availability

Raw sequence reads are available in the NCBI Sequence Read Archive under BioProject ID PRJNA1299142.
