# Supplementary figures and images for: Bacterial Communities Associated With Acute Oak Decline of Sessile Oak ( Quercus petraea ) in Southern Sweden
Source: Environ Microbiol Rep. 2025 Nov 21;17(6):e70244. doi: 10.1111/1758-2229.70244 (PMC12638204; doi:10.1111/1758-2229.70244)

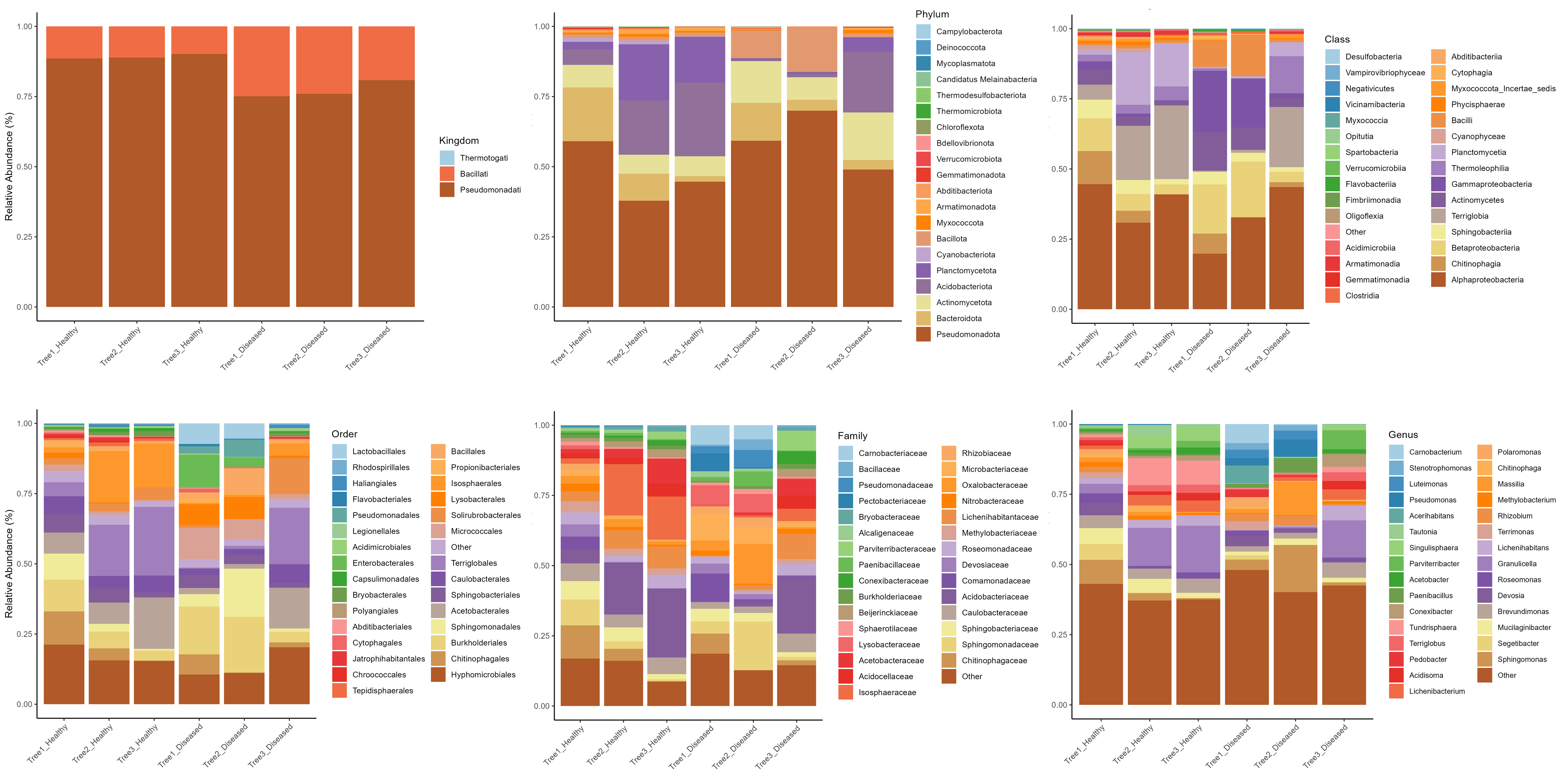

Supplement: Supplementary file 1 — Figure S1: Column plots show relative abundance of healthy and damaged samples per oak tree individual at different taxonomic levels. The plots show the 30 most abundant taxa and all the others referenced as the group “Other”. [file EMI4-17-e70244-s001.png]

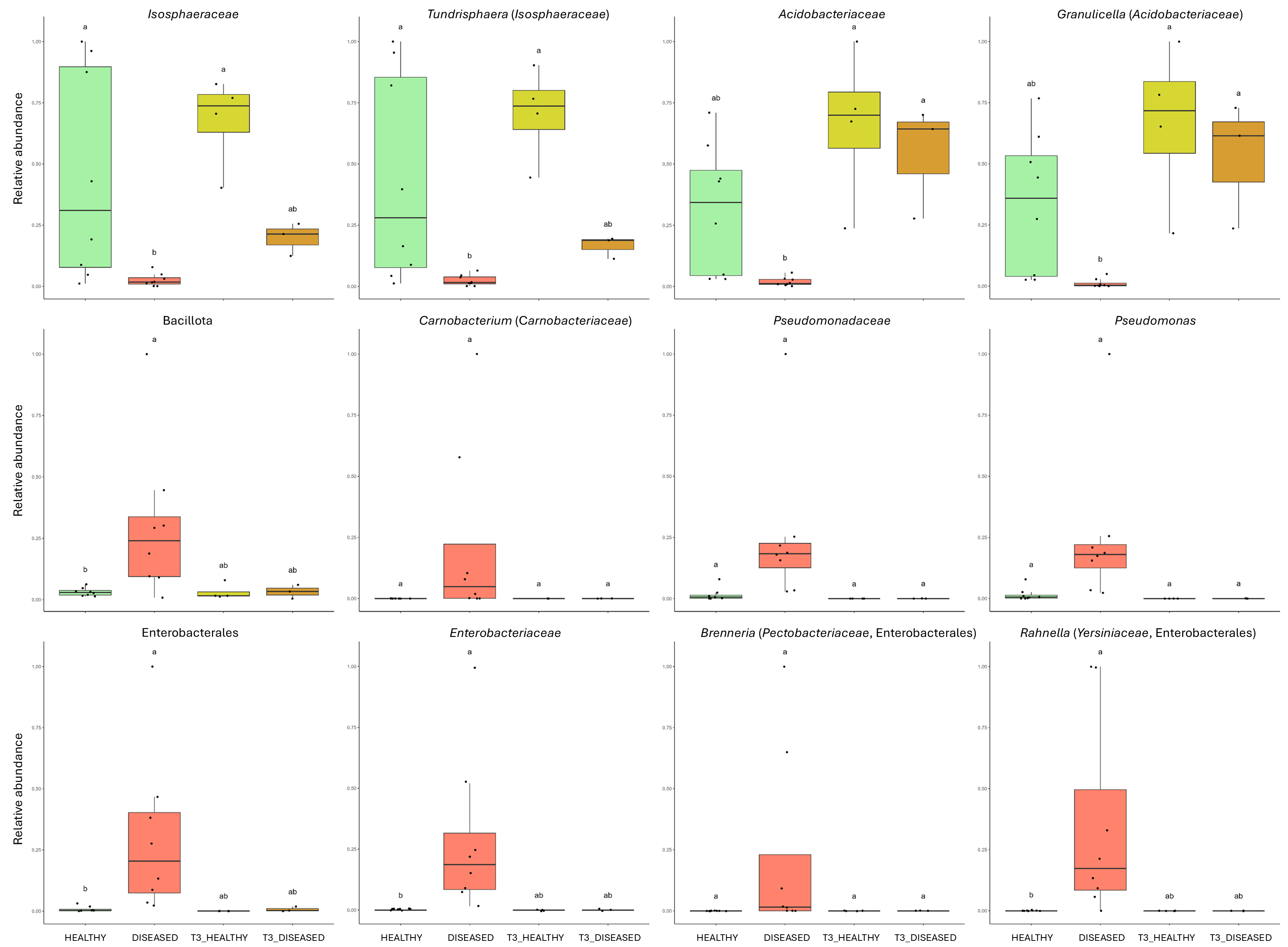

Supplement: Supplementary file 2 — Figure S2: Boxplots showing the relative abundances of selected bacterial taxa across the four oak bark sample groups: healthy bark samples (light green), diseased bark samples (pale red), healthy bark tissues of Tree_3 (dark yellow), diseased bark tissues of Tree_3 (dark orange). Each plot represents one bacterial taxon, with individual data points overlaid on boxplots. Statistical differences amongst groups were assessed using one‐way ANOVA followed by Tukey's honest significant difference (HSD) post hoc test. Different letters above the boxes indicate statistically significant differences between groups (p < 0.05). [file EMI4-17-e70244-s002.png]
